# Spectroscopic Analysis of the Complex Refractive Indices for Imine Brown Carbon Aerosol Particles

**DOI:** 10.1021/acsearthspacechem.5c00392

**Published:** 2026-02-12

**Authors:** Simon Xi Chen, Gwen R. Lawson, James D. Allan, Justin M. Langridge, Michael I. Cotterell

**Affiliations:** 1 School of Chemistry, 1980 University of Bristol, Bristol BS8 1TS, U.K.; 2 Department of Earth and Environmental Sciences, 5292 University of Manchester, Manchester M13 9PL, U.K.; 3 National Centre for Atmospheric Science, The University of Manchester, Manchester M13 9PL, U.K.; 4 11365 Met Office, Exeter EX1 3PB, U.K.; 5 Department of Chemistry, University of Oxford, Oxford OX1 3QZ, U.K.

**Keywords:** brown carbon, glyoxal, refractive index, spectroscopy, aerosol pH, aerodynamic particle selection

## Abstract

Brown carbon (BrC) contributes substantially to light absorption by atmospheric aerosols, which represents a key uncertainty in estimates of aerosol radiative forcing. Among its diverse constituents, imine BrC formed from aqueous reactions between glyoxal and nitrogen-containing species is of widespread interest. We report sensitive measurements of particle size-resolved optical cross sections, associated retrievals of complex refractive indices (*m* = *n* + *ik*), and effective density measurements for imine BrC aerosols generated from aqueous solutions containing glyoxal and nitrogen species across a wide pH range. An Aerodynamic Aerosol Classifier was coupled with cavity ring-down and photoacoustic spectroscopy to measure extinction and absorption cross sections at the short-visible wavelength of 405 nm, enabling retrievals of *n* and *k* with high precision and accuracy while eliminating multiply charged particle artifacts that impair more common mobility-based approaches. Detectable light absorption was observed under basic conditions (pH ≈ 9.5) only, yielding values of *k* in the range 0.0016–0.0018 and *n* of ∼1.50, demonstrating that imine BrC may contribute modest absorption at short-visible wavelengths. Meanwhile, aerosols generated from aqueous solutions under acidic and near-neutral conditions were nonabsorbing (*k* < 10^–4^). Comparison with bulk UV/vis absorption spectra indicates that aerosolization and drying enhanced chromophore formation under basic conditions, likely through the supersaturation of dissolved reacting solutes in drying aqueous aerosol droplets. Measured values for effective particle density were consistent with the formation of partially oligomerized glyoxal hydrates.

## Graphical abstract

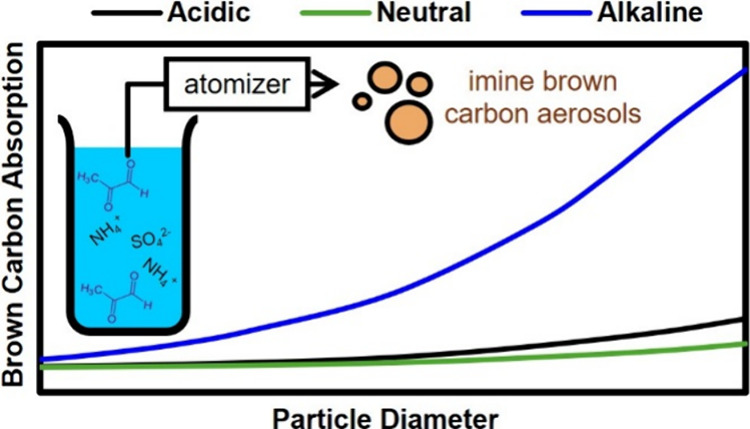

## 1 Introduction

Aerosols exert a net cooling effect on Earth’s surface temperature, yet significant uncertainties persist in quantifying their precise contributions to radiative forcing including from aerosol-radiation interactions.^1^ These uncertainties are especially pronounced for light-absorbing particles, which represent one of the largest sources of uncertainty in projecting climate. Progress toward reducing these uncertainties requires new accurate and precise measurements of the optical properties for these light-absorbing aerosol particles.

Patterson and McMahon identified distinct optical properties between aerosols emitted during flaming and smoldering phases of biomass combustion.^2^ Flaming-phase emissions produced black particles with a strong visible-spectrum absorption and weak wavelength dependence, while smoldering-phase emissions yielded light-brown particles with significant wavelength-dependent absorption. Mukai and Ambe detected atmospheric brown particles hypothesized to originate from smoldering vegetation.^3^ These findings contributed to the conceptualization of brown carbon (BrC).^4^ BrC is now recognized as a class of organic aerosols characterized by weak light absorption at mid- to long-visible wavelengths but sharply increasing absorption toward short-visible and ultraviolet (UV) regions.^4–8^

BrC contributes substantially to atmospheric light absorption, accounting for 15–50% of total absorption across the atmosphere, snow, and sea ice.^6,9–12^ Estimates of BrC radiative forcing vary by a factor of ∼ 15, from approximately 0.04 to 0.57 W m^–2^.^12,13^ This large variation derives from uncertainties in BrC optical properties, and how these evolve over particle lifetime.^12^ BrC species found in the atmosphere include polyaromatic hydrocarbons, oligomerized aldehydes, nitroaromatics, and imines. This work focuses on imines, which form in the aqueous phase through the reaction of nitrogen-containing species (such as NH_3_, (NH_4_)_2_SO_4_, and amines) with dicarbonyl species, particularly glyoxal and methylglyoxal.^14–17^ Glyoxal and methylglyoxal are estimated to have atmospheric production rates of 45 Tg annum^–1^ and 140 Tg annum^–1^, respectively, with the oxidation of isoprene (a species of biogenic origin) contributing to 47% of glyoxal and 79% of methylglyoxal loadings globally.^18^ The produced imines are diverse in composition and principal components are imidazole and its derivatives,^14–17^ particularly imidazole-2-carboxaldehyde.^17^ The rates of imine formation are slow in laboratory experiments on bulk solutions, with reaction time scales of ∼ weeks and require high reactant concentrations on the order of ∼ 1 M.^15,19^ However, imine formation may proceed with enhanced rates when these reactions occur in aerosols compared to reactions in macroscopic bulk solutions, likely driven by the ability of aerosols to access metastable supersaturated solute concentration states as water is removed during humidity cycling (e.g., cloud processing), with such concentrations inaccessible in bulk solutions.^14,20^ Laboratory studies also show that the reactive uptake of glyoxal vapor on ammonium sulfate aerosol particle surfaces occurs on time scales of minutes.^21–24^ This surface-mediated formation of imines becomes an important pathway in aerosols given their high surface-to-volume-ratios. Teich et al. analyzed environmental aerosol samples and identified various imidazole species at concentrations in the range 0.2–14 ng m^–3^.^25^

Quantifying the radiative forcing of BrC requires accurate descriptions of its complex refractive index (*m* = *n* + *ik*). The real component of the refractive index (*n*) specifies the phase speed of light in a material, while the imaginary component (*k*) characterizes light absorption by the material comprising an aerosol particle.^26^ The values for *m* are key determinants of the extinction (σ_ext_), scattering (σ_sca_), and absorption (σ_abs_) cross sections of a particle, which are described accurately by Lorenz-Mie theory for spherical homogeneous particles.^27,28^ These cross sections are critical for calculating properties such as single-scattering albedo that are input to radiative forcing calculations.^27,28^

Values for *n* and *k* may be determined for aerosol particles by comparing measurements of their optical cross sections to Lorenz-Mie theory calculations. Aerosol refractive indices have been retrieved from such comparisons with extinction cross sections, with aerosol extinction measured using cavity ring-down spectroscopy (CRDS).^19,23,29–32^ Although CRDS provides sensitive measurements of aerosol extinction cross sections, extinction-only retrievals of *n* and *k* suffer from high levels of uncertainties, and concurrent measurements of an additional (absorption or scattering) cross section are needed to reduce these uncertainties.^33^ Photoacoustic spectroscopy (PAS) has emerged as a robust and accurate approach to measuring absorption by nonvolatile aerosol particles with submicrometer diameters.^34–39^ Combining CRDS-measured extinction cross sections and PAS-measured absorption cross sections enables accurate and precise determinations of aerosol complex refractive indices.^33,38^

The refractive index retrieval approach takes measurements of cross sections at multiple values of aerosol particle size and compares the measured size-dependent cross section distributions to those predicted by Lorenz-Mie theory. However, a critical challenge lies in the selection and control of particle size. Differential mobility analyzers (DMAs), used widely for mobility-based particle size selection, introduce systematic errors in retrieved refractive indices deriving from multiply charged particles.^40^ For example, Khalizov et al. reported scattering cross sections 17–47% higher than theoretical predictions for ammonium sulfate due to these charge artifacts.^41^ Although methods to minimize multiply charged fractions exist, they often reduce aerosol particle number concentrations thereby compromising measurement sensitivity, and do not remove completely the multiply charged particles.

Recent advances in aerodynamic aerosol classification offer a new approach to the selection and control of particle size. The aerodynamic aerosol classifier (AAC), developed by Tavakoli and Olfert,^42^ selects particles on their relaxation time (which is connected directly to the particle aerodynamic diameter) without requiring prerequisite charging of the aerosol sample.^43^ Moreover, the AAC achieves up to 5-fold higher transmission efficiency than DMAs,^44^ enabling higher particle concentrations and improved precision in downstream optical measurements. We recently used the AAC to size-select dried aerosol particles, with downstream CRDS and PAS instruments characterizing their optical properties in addition to a Scanning Mobility Particle Sizer (SMPS) and Condensation Particle Counter (CPC) for measuring the particle mobility size distribution. This approach was applied successfully in the Soot Aerodynamic Size Selection for Optical properties (SASSO) campaign to characterize the optical cross sections and refractive indices for soot particles,^45,46^ and we assessed rigorously the improved precision and accuracy of aerosol optical property measurements and associated refractive index retrievals when using an AAC rather than a DMA.^39^

This work reports measurements of the complex refractive indices for laboratory-generated imine BrC aerosols under tightly regulated aerosol generation and measurement conditions, using our AAC-selection approach coupled with downstream optical and mobility particle sizing measurements. We focus on imine BrC formed from the reaction of glyoxal with nitrogen-containing species inside drying aerosol particles.

Zhou et al. measured concurrently the extinction and scattering cross sections of dried imine BrC aerosol particles generated from prereacted (for 14 days) aqueous solutions containing glyoxal or methylglyoxal mixed with ammonium sulfate, glycine, or dimethylamine.^47^ The dried particles were size-selected using a DMA prior to measurements of their extinction and scattering cross sections: CRDS measurements of extinction were performed at a wavelength (λ) of 532 nm and integrating nephelometry measurements of scattering were performed at wavelengths of 450, 525, and 635 nm that were interpolated to λ = 532 nm. The pH of the solutions in their study was highly variable, ranging from 1.77 to 12.67. Nonetheless, the retrieved values for *k* (λ = 532) for the aerosols were remarkably similar, with all samples having *k* values of ∼ 0.04 that correspond to large levels of light absorption at this midvisible wavelength. Such large values of *k* are unexpected given that these imine BrC chromophores exhibit absorption bands peaking at UV wavelengths <300 nm, and the retrieved *k* values may have been biased high from the correction of the nephelometry measurements to λ = 532 nm, inaccuracy in the required scattering truncation correction, and the role of multiple charge artifacts from DMA selection. Marrero-Ortiz et al. examined the refractive index of glyoxal- and methylglyoxal- mixtures with amines, using a photoacoustic extinctiometer to concurrently measure extinction and absorption for mobility-selected particles.^48^ They reported *k* (λ = 405 nm) in the range 0.002–0.010 for glyoxal-amine mixtures, although the measured absorption cross sections are reproduced poorly by their best-fit calculations.

Here, we report measurements on imine BrC samples similar to those analyzed by Jansen and Tolbert,^15^ with our work providing measurements of the complex refractive index of the generated particles that were not reported in this previous study. Jansen and Tolbert measured the optical properties of imine BrC aerosol particles generated from prereacted aqueous solutions containing glyoxal and either ammonium sulfate or ammonia.^15^ The aqueous precursor solutions contained dissolved glyoxal and different amounts of ammonium, ammonia, sulfuric acid, and sodium sulfate and were left for 3–4 months to react. CRDS and PAS were used to characterize the optical properties of the dried aerosol particles at spectroscopic wavelengths of 405 and 532 nm. Prior to spectroscopic characterization, the aerosol ensembles were not size-selected, and instead the full distributions were passed to CRDS and PAS spectrometers and an SMPS. Consequently, measurements of aerosol optical properties relied on quantifying a mass absorption cross section rather than refractive indices that represent better, intensive metrics for optical properties. For aerosol particles generated from these solutions, those with a pH of ∼ 4 demonstrated the smallest mass absorption cross sections (MAC, with values ∼ 3 × 10^–5^ m^2^ g^–1^), and the absorption increased regardless of whether the initial solution from which the aerosol particles were generated became more acidic or basic. The authors reported the highest absorption for particles corresponding to the most acidic (pH ∼ 0.7, with MAC ∼ 2.2 × 10^–4^ m^2^ g^–1^) and the most basic (pH ∼ 8.4, with MAC ∼ 2.7 × 10^–4^ m^2^ g^–1^) solutions. To characterize refractive index, our experimental design used an AAC upstream of our CRDS-PAS measurement system. This approach enabled size-resolved optical cross section measurements by segregating particles into low-polydispersity and monomodal populations prior to analysis, using the same analysis approach described by Lawson et al.^39^ By comparing the size-dependent cross sections with predictions from Lorenz-Mie theory calculations, we retrieved the complex refractive indices of our aerosol particles. The measurement approach also allows for determinations of the effective mass densities of the sampled aerosol particles.

The following section describes our experimental methods for analyzing bulk solution and aerosol properties. Section 3 presents analysis of our measurements on imine BrC aerosols formed from the reactions of glyoxal with nitrogen containing species in different pH environments. Section 4 summarizes our work and suggests future research avenues.

## 2 Experimental Methods

### 2.1 Preparation of Imine BrC Solutions

Aerosol particles were generated from imine BrC solutions with identical compositions to those analyzed by Jansen and Tolbert.^15^ Table 1 summarizes the molar concentrations of imine BrC solutions that were prepared and are denoted as vial A, E, and K to match the nomenclature used by Jansen and Tolbert. The pH of the solutions was varied from 0.69 to 8.43 by addition of controlled amounts of ammonia or sulfuric acid, and each solution contained 1 M glyoxal. We prepared two sets of solutions: one set of fresh solutions were used to generate aerosol particles immediately after preparation; a second set of aged solutions were stored in dark conditions to limit exposure to ambient light and left to react over the course of 1 month prior to aerosol particle generation. The pH values for the ‘fresh’ and ‘aged’ solutions, before and after 50-fold dilution, were measured (with a measurement uncertainty of 0.1 units) by a pH meter (HANNA, HI9811–5), and referred to as pH_fresh.conc_, pH_fresh,dil_, pH_aged,conc_, and pH_aged,dil_ to denote the pH for fresh concentrated, fresh diluted, aged concentrated, and aged diluted solutions, respectively. The factor of 50 dilution was performed to replicate the sample generation procedure of Jansen and Tolbert.^15^

**Table 1:** Initial Chemical Concentrations (mol dm^–3^, M) of Vial A, E, and K^a^

	(NH_4_)_2_SO_4_	NH_3_	H_2_SO_4_	Na_2_SO_4_	glyoxal	pH_fresh,conc_	pH_fresh,dil_	pH_aged,conc_	pH_aged,dil_
A	0.501		0.515		1.004	0.5	1.9	0.7	1.9
E	0.500			0.501	1.004	3.1	3.7	2.4	3.3
K		0.999		1.000	1.001	9.5	9.3	5.9	5.7

a The pH values measured for the initial fresh (denoted with a subscript ‘fresh’) and aged (denoted with a subscript ‘aged’) solutions before (pH_fresh,conc_ and pH_aged,conc_) and after dilution (pH_fresh,dil_ and pH_aged,dil_) are tabulated.

### 2.2 UV/Vis Absorption Spectroscopy Analysis of Bulk Solutions

UV/vis absorption spectra were measured for the fresh solutions under 50-fold dilution. For comparison, the aged solutions were measured for both concentrated and at 50-fold dilution. Additional measurements for 200-fold diluted solutions were used for some aged samples that, at 50-fold dilution, exceeded the transmission detection limit of our UV instrument. Small aliquots of the solutions were pipetted into 1 cm path length Hellma quartz cuvettes (100–10–46), and their absorption spectra recorded using a UV/vis spectrometer (Agilent, G6860A). Background spectra were recorded for the cuvettes filled with water. The measured absorbance, *A*, for a solution was converted to an imaginary refractive index spectrum, *k*_soln_, using

$$k_{\mathrm{soln}}=\frac{\lambda A\ln\left(10\right)}{4\pi l}$$ 1

in which λ is the wavelength, and *l* is the optical path length through the cuvette (1 cm). *k*_soln_ is connected to the imaginary refractive indices of the individual components of the mixture via the linear mass fraction weighting mixing rule:^49^

$$k_{\mathrm{soln}}=k_{H_{2}O}w_{H_{2}O}+k_{\mathrm{solute}}w_{\mathrm{solute}}$$ 2

in which *k*_H_2_O_ and *k*_solute_ are the imaginary refractive indices for water and solute, and *w*_H_2_O_ and *w*_solute_ are the mass fractions of water and solute, respectively, which are known from the measured solute masses and dilution factors. The value for *k*_H_2_O_ is <10^–7^ and is negligible. Moreover, the background spectrum that was subtracted from measured spectra corresponded to that of the cuvette filled with water, and therefore any absorption features attributed to water were removed when calculating the spectra shown in Section 3. Therefore, assuming *k*_H_2_O_ to be zero, *k*_solute_ was calculated using

$$k_{\mathrm{solute}}=\frac{k_{\mathrm{soln}}}{w_{\mathrm{solute}}}$$ 3

### 2.3 Spectroscopic Analysis of Aerosol Particles

Our method for spectroscopic analysis of aerosol particles is identical to that described in our previous publication.^39^ We refer the reader to this previous publication for a full description of the spectrometers used and a comprehensive assessment of the accuracy and precision of our optical cross section measurements and retrieved complex refractive indices. Figure 1 summarizes our approach to aerosol generation, conditioning, particle size selection, and downstream aerosol characterization. Our experiments used CRDS and PAS to measure the extinction and absorption coefficients, respectively, at λ = 405 nm, with the PAS spectrometers calibrated using ozone-laden gas samples.^50–52^ Combined with concurrent CPC measurements of the particle number concentration, the ensemble-mean single-particle extinction and absorption cross sections were determined. A novel feature of our methodology is the use of an AAC to enable accurate and precise characterizations of the particle size dependence to optical cross sections for subsequent retrievals of aerosol particle complex refractive indices.

**Figure 1:**
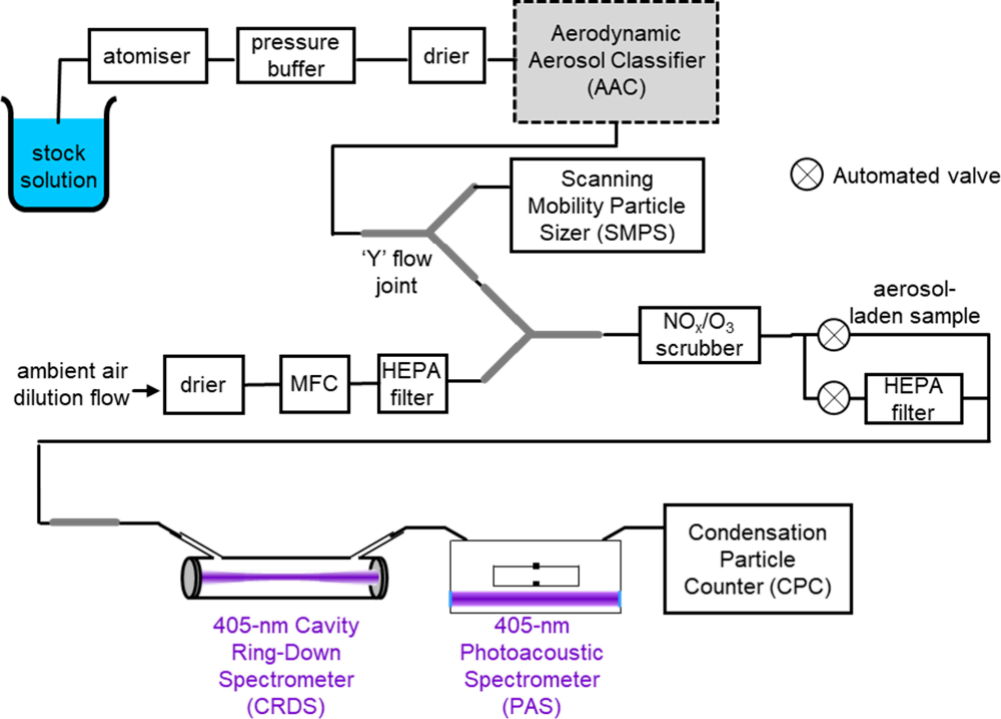
Experimental configuration used for aerosol generation, conditioning, particle size selection, and downstream characterization of the aerosol samples using a CRDS, PAS, SMPS, and CPC. MFC denotes a mass flow controller, HEPA denotes a High Efficiency Particulate Air filter.

Aqueous aerosol particles were generated in air using a TSI 3073 Portable Test Aerosol Generator from 50 mL volume solutions. The aerosol-laden sample was then passed through two Cambustion diffusion drier units to reduce the relative humidity (RH) of the sample below 5%. The dried particles were drawn through the AAC that selected aerosol particles on their aerodynamic diameter, prior to the particles being interrogated using CRDS for measurements of aerosol extinction coefficient (α_ext_), PAS for measurements of aerosol absorption coefficient (α_abs_), and a CPC (TSI 3756) for measurements of aerosol particle number concentration (*N*). The wavelength of the laser for extinction and absorption measurements was 405 nm, with separate lasers used for the CRDS and PAS spectrometers. Combining measurements of these optical coefficients and number concentrations, the ensemble-mean extinction (σ_ext_) and absorption (σ_abs_) cross sections were determined, by dividing the measured coefficients (α_ext/abs_) by the particle number concentration:

$$\sigma_{\mathrm{ext}/\mathrm{abs}}=\frac{\alpha_{\mathrm{ext}/\mathrm{abs}}}{N}$$ 4

The particle mobility size distributions of the AAC-selected particles were measured by an SMPS comprised of an electrostatic classifier (TSI 3082), a 3088 soft X-ray neutralizer, a 3081 long differential mobility analyzer, and a CPC (TSI 3789). These measurements of ensemble-mean cross sections and mobility size distributions were repeated for 13 values of aerodynamic diameter between 100 and 400 nm in 25 nm intervals, enabling characterizations of the dependence of the optical cross sections on particle size. The resolution parameter (*R*_s_) of the AAC, which represents the ratio of the set aerodynamic diameter to the full width at half-maximum of the transfer function, was set to 20.

The real and imaginary refractive index of the particles were retrieved from the particle-size dependent measurements of the extinction and absorption cross sections. We used trial values for the *n* and *k* components of the refractive index and the mobility diameter distributions measured by the SMPS as inputs to a Lorenz-Mie calculation of the ensemble-mean extinction and absorption cross sections. Implicitly, this Lorenz-Mie modeling approach assumes that the sampled aerosol particles exhibit spherical shapes and homogeneous compositions. Section 3.3 demonstrates excellent agreement between measured and modeled optical cross sections, validating our choice of optical modeling approach. A merit function quantified the level of agreement between experimentally measured and modeled cross sections across all selected aerodynamic diameters, and *n* and *k* were varied using a grid search algorithm to minimize the value of the merit function. Complex refractive indices were retrieved by simultaneously fitting the extinction and absorption data. We refer the reader to Lawson et al. for full details of the treatment of the mobility size distribution, and the fitting procedure used to optimize the fit of Lorenz-Mie cross section distributions to measured values.^39^

## 3 Results and Discussion

### 3.1 pH Measurements for Imine BrC Solutions

Figure 2 compares our measurements of imine bulk solution pH values with those reported by Jansen and Tolbert (pH_lit_) for their freshly prepared concentrated solutions.^15^ Our measured values for pH_fresh,conc_ compare well with pH_lit_, with differences up to ∼ 1 pH unit possibly caused by differences in the calibration accuracies of the pH meters in the two studies, the pH of the water used in solution preparation, and (in the case of vial K) varying degrees to which ammonia evaporated between solution preparation and pH measurement. The aged vial K solution was considerably less basic than when freshly prepared, attributed to evaporative loss of ammonia, while the pH of vial A and E exhibited negligible change with aging. Dilution by a factor of 50 had the expected effect of shifting the pH of the solutions by a small amount toward more neutral conditions. Jansen and Tolbert also measured the pH of aqueous aerosol droplets generated from their solutions using the technique from Craig et al.^15,53^ Briefly, aerosol particles were impacted onto pH indicator paper immediately after atomization at an RH of 80–84%, and photographs of the pH paper were taken and combined with colorimetric analysis to determine the pH. Despite a reduction in pH by up to two units at near-neutral conditions, their results demonstrated that the pH for the most acidic and most basic samples were nearly identical for the bulk and aerosol samples immediately after atomization.

**Figure 2:**
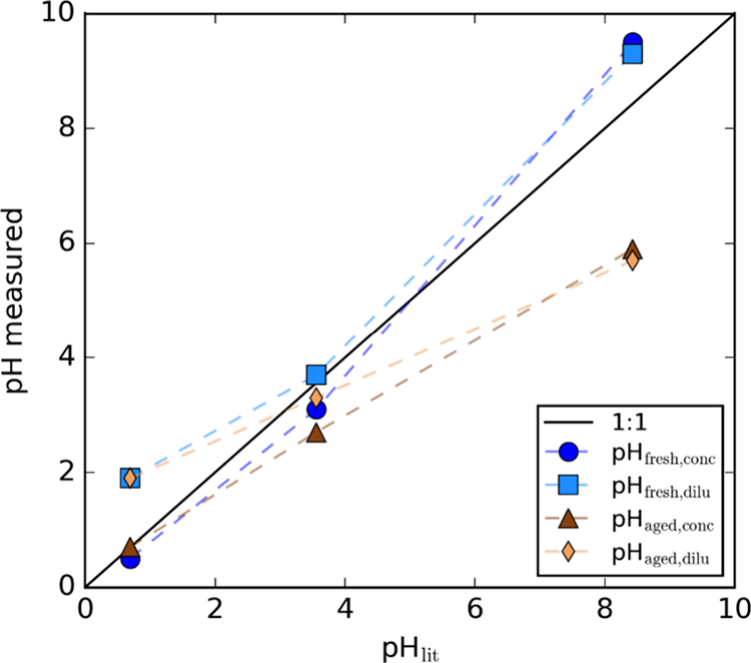
Comparison of measured pH (including pH_fresh,conc_, pH_fresh,dil_, pH_aged,conc_, and pH_aged,dil_) with those reported by Jansen and Tolbert (2023) (pH_lit_). The solid black line represents the 1:1 ratio between these two values. The dashed lines are to guide the eye only.

### 3.2 UV/Vis Absorption Measurements for Imine BrC Bulk Solutions

The absorption spectra of the aged solutions under concentrated and 50-fold dilution conditions often demonstrated absorbances that exceeded values of 3.2, outside of the measurement range for our UV/vis spectrometer (Figure S1 in the Supporting Information). Therefore, we performed additional measurements on solutions diluted 200-fold to provide absorbance data over the 200–800 nm wavelength range for aged solutions. Figure 3 shows the *k*_solute_ spectra corresponding to the solute, determined from the measured absorbances using eqs 1 and 3. The imaginary component of the refractive index is an intensive optical property independent of the solute concentration in the aqueous solution interrogated by the UV/vis spectrometer.^26^ Some spectra are missing data toward higher *k*_solute_ values, which arises because of the measured absorbance exceeding 3.2 as described above. Figure 3 shows that the *k*_solute_ values are comparable for the aged solutions with different dilution factors, as expected if the linear mass fraction mixing rule (eq 2) holds. We note that our *k*_solute_ calculations assumed the same solute mass fractions added to the initial freshly prepared aqueous solutions. However, as indicated in Figure 2 and explored further in Section 3.4, ammonia has likely evaporated from the vial K reaction mixtures with aging and therefore the solute mass fractions reduced; repeating the *k*_solute_ calculations under the assumption of complete evaporation of ammonia for the aged vial K solutions leads to larger values than indicated in Figure 3 by up to 7%. Jansen and Tolbert reported that basic conditions favored brown carbon formation in bulk solutions,^15^ and our results in Figure 3 show similar trends, with *k*_solute_ for aged vial K solutions showing a considerable increase at sub-500 nm wavelengths, indicating brown carbon formation at the most basic conditions explored in our work. The fresh vial K spectrum shows only slight increases in *k*_solute_ at sub-350 nm wavelengths. These slight increases indicate that the bulk reaction of glyoxal with ammonia proceeded at a rate fast enough to produce light-absorbing imine products on the subhour time scale between solution preparation and the UV/vis absorption spectroscopy measurement. This observation is consistent with those from Marracci and Murray,^14^ who reported the bulk reaction formation of imine BrC in aqueous solutions containing glyoxal and ammonium sulfate at concentrations of 0.42 mol dm^–3^ and 2.70 mol dm^–3^, respectively, with detectable increases in imidazole concentrations after ∼ 2 h using their microdroplet Raman method. The absorption band centered at the wavelength of ∼ 280 nm is consistent with reported absorption spectra for imidazole-2-carboxaldehyde and 4-methyl-imidazole-2-carboxaldehyde.^54^ At the 405 nm wavelength pertaining to our CRDS and PAS spectroscopy measurements, *k*_solute_ is negligible and less than 10^–4^ for the reactions occurring at pH ∼ 1 (vial A) and pH ∼ 3 (vial E) for both aged and fresh solutions. For the reaction occurring under the most alkaline conditions explored, the aged vial K solutions exhibited *k*_solute_ values at the 405 nm wavelength in the range 0.0007–0.0009 and are much larger than that for the fresh vial K solution for which *k*_solute_ is negligible.

**Figure 3:**
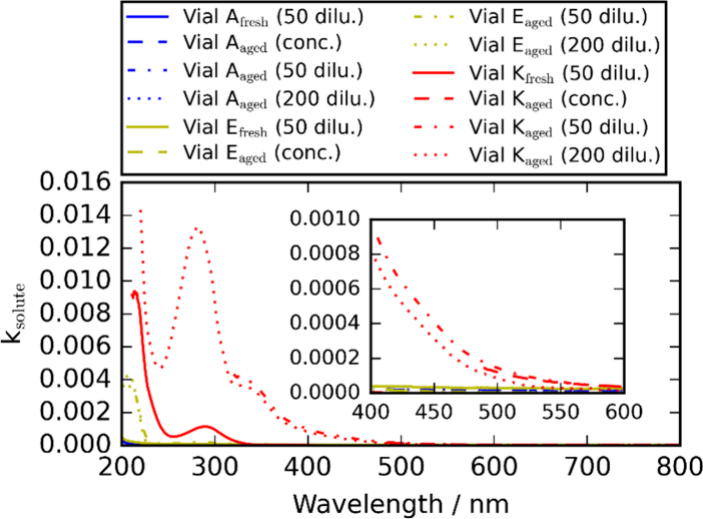
Determined wavelength-dependent imaginary component of the refractive index of the solute, *k*_solute_, for vial A, E, and K, for the 50-fold diluted fresh solutions as well as aged solutions under concentrated, 50-, and 200-fold dilution conditions.

### 3.3 Optical Characterization of Imine BrC Aerosols

We characterized the optical properties of aerosol particles generated from the diluted solutions, corresponding to vial A, E, and K, using the approach described in Section 2.3. Optical attenuation coefficients, particle number concentrations, and particle mobility size distributions were recorded at each AAC-selected diameter for 5 min. For all measurements, the recorded light absorption coefficient at each AAC-selected diameter was small (<1 × 10^–5^ m^–1^). Indeed, Jansen and Tolbert did not utilize size selection in their work and instead sampled the entire aerosol plume after atomization and drying with their CRDS and PAS spectrometers,^15^ possibly to enable absorption coefficient measurements above the sensitivity level of their PAS instrument.

The properties of the bulk aged solutions, and the aerosol particles generated from them, were compared to optical measurements on aerosols generated from fresh solutions to investigate whether the reactions to form imine BrC were accelerated by aerosolization and whether the chromophores in the bulk solution remained in the condensed (particle) phase following aerosolization and drying. The fresh solutions were used immediately after preparation to generate aerosol particles, and we append the subscript labels ‘fresh,conc’ and ‘fresh,dil’ to indicate properties attributed to concentrated and diluted forms of these freshly prepared solutions, respectively. We append the subscript labels ‘aged,conc’ and ‘aged,dil’ to properties attributed to aerosols generated from the concentrated and diluted forms of the ∼ 1-month aged solutions, respectively. The fresh solutions were diluted (by a factor of 50) to limit any reactions in the bulk solutions and for consistency with the previous measurements by Jansen and Tolbert.^15^ The aged solutions were also diluted by a factor of 50 to enable comparisons of the determined optical properties with those generated from the fresh solutions and allow assessments of any reaction acceleration induced by aerosolization and prerequisite drying prior to spectroscopic analysis. Aerosols generated from atomization of the ∼ 1-month aged solutions in their concentrated form were also analyzed to enable assessments of any dilution effects on solute composition, e.g. through the impacts of dilution on bulk solution pH that might affect molecular compositions and chromophore concentrations.

For all data sets, Figures S2 and S3 in the Supporting Information compare the measured extinction and absorption cross sections with their corresponding best-fit Lorenz-Mie theory predictions. Figure 4 shows a selection of this data, showing the variation in the cross sections with the selected aerodynamic diameter for the aged, concentrated solutions. The standard deviations in the calculated cross sections were determined through an error propagation of the standard deviations in the measured mean optical coefficients and number concentrations. The large standard deviations indicated for some absorption cross sections stem from the low absorption coefficients for those aerosol samples that have magnitudes close to the sensitivity limit of our 405-nm PAS spectrometer. The cross section data clearly demonstrate the high precision in the measured cross sections and low magnitudes of the measured σ_abs_ in comparison to those for σ_ext_, with σ_ext_ over 2 orders of magnitude larger than σ_abs_. The σ_abs_ data in Figure 4 show that statistically meaningful levels of absorption were detected for vial K only, indicating that imine formation is only significant for the most basic conditions explored in this work.

**Figure 4:**
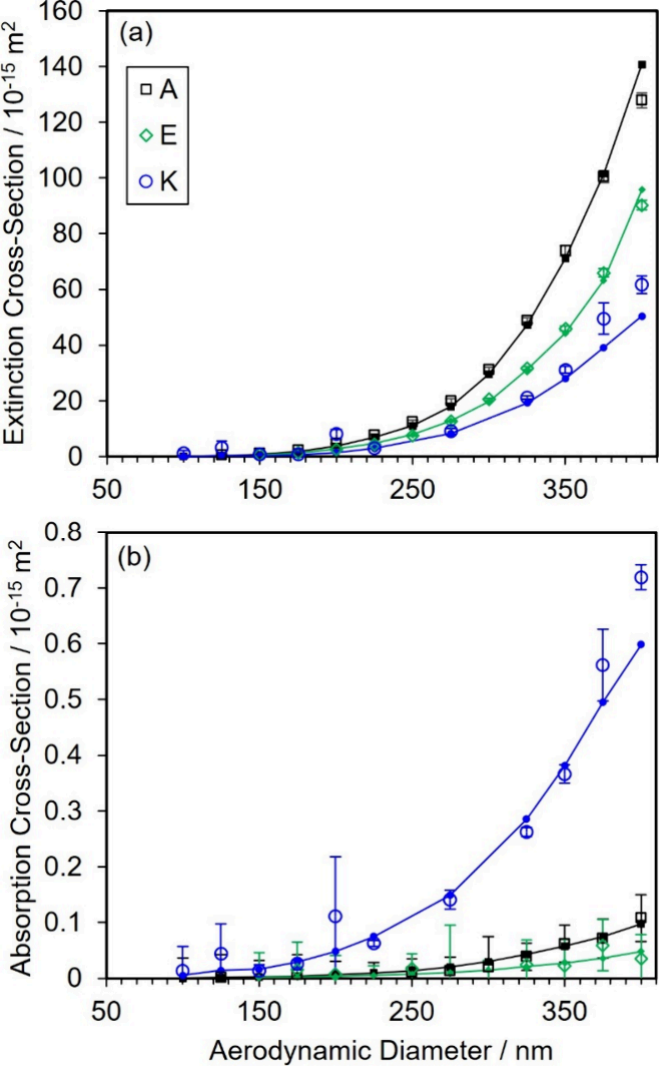
For the aged, concentrated solutions, the measured and modeled optical cross sections with variation in the selected aerodynamic diameter are compared for: (a) extinction cross sections; (b) absorption cross sections. Open symbols denote measured values, and filled symbols denote the best-fit cross sections from Lorenz-Mie theory. Error bars represent one standard deviation in the measured cross sections. Lines are to guide the eye only for the best-fit modeled cross sections. Black, green, and blue data points correspond to vial A, E, and K, respectively.

Figure 5 shows the variation in the retrieved refractive indices (also tabulated in Table S1) with pH_fresh,conc_. We opt to show the *n* and *k* as functions of pH_fresh,conc_ such that solutions with the same starting compositions are more easily compared and trends in *n* and *k* for vials A, E, and K with aging and dilution are easier to visualize, but the changes in bulk solution pH identified in Section 3.1 should be kept in mind. Aerosols were interrogated for those corresponding to aged solutions in both concentrated and diluted forms, and the imaginary refractive indices from these aerosol measurements are compared with values from our UV/vis absorption spectroscopy analysis on bulk solutions (i.e., the values from Figure 3 that correspond to λ = 405 nm). Given that the pH of the samples were not altered appreciably by dilution (see Section 3.1), Figure 5 demonstrates a high level of reproducibility in retrieved values for *k* for aerosol particles generated from the aged solutions under both concentrated and 50-fold dilution conditions. However, the retrieved *n* are more variable for the corresponding vial A (pH_fresh,conc_ = 0.5) or E (pH_fresh,conc_ = 3.1) samples. The retrieved *n* is 1.491 and 1.546 for the aerosol particles generated from concentrated and diluted solutions of vial A, respectively, and are 1.532 and 1.482 for particles generated from concentrated and diluted solutions of vial E, respectively. In both cases, the causes for these differences in *n* of ∼ 0.05 between particles are unclear. Indeed, much of this difference in *n* is reconciled by the uncertainty in the retrieved *n* that was assessed previously to be ± 0.02.^39^

**Figure 5:**
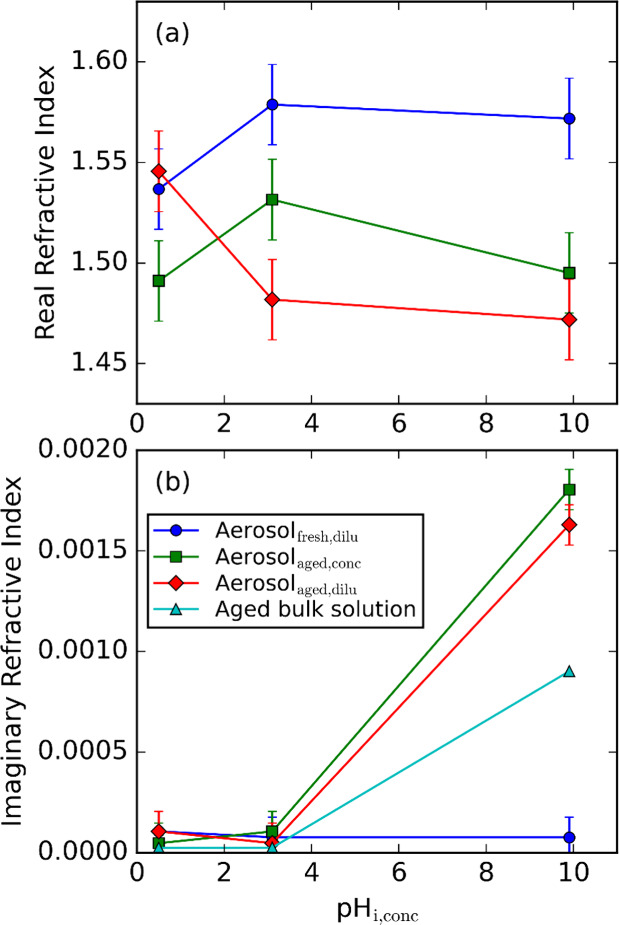
(a) Real (*n*), and (b) imaginary (*k*) components of the refractive index for imine BrC samples as a function of pH_fresh,conc_. The lines are to guide the eye only. Error bars represent uncertainties of ± 0.02 for *n* and ± 0.0001 for *k*, assessed previously by Lawson et al.^39^

Our UV/vis measurements on bulk solutions (Section 3.2) did not observe any formation of light absorbing chromophores in the visible spectrum for the vial A or E reaction mixtures. For the aerosol particles generated from aged solutions, we did not observe an increase in *k* in acidic conditions in contrast to the observations of Jansen and Tolbert (although we note the increase in absorption reported by those authors at the most acidic conditions was observed for a single repeat only),^15^ but non-negligible values for *k* are determined for the most basic conditions studied. Considering the aged samples of vial K (pH_fresh,conc_ = 9.5), the imaginary refractive index from our aerosol analysis is almost twice that determined from our bulk UV/vis measurements. This difference in *k* is not reconciled by considering the aforementioned evaporation of ammonia from the solution (which will increase the *k*_solute_ value determined from our UV/vis analysis by up to ∼ 7%). Instead, the enhanced absorption in the aerosol measurements may indicate the accelerated formation of chromophores via aerosolization, possibly from the enhanced concentration of reacting species as the particles undergo drying and transition to their dried forms via metastable states of supersaturated solute concentrations, similar to previous observations.^14,20^ Such absorption enhancement is not observed for aerosol particles generated from the freshly prepared solutions. For these particles, the retrieved *k* do not show any detectable changes with pH_fresh,conc_ and values remain close to the sensitivity threshold of the retrieval and within statistical agreement at all pH values.

Our *k* values at λ = 405 nm are approximately 1 order of magnitude lower than those reported by Zarzana et al. at a much longer wavelength of λ = 532 nm, with their *k* values ranging from 0.035 (±0.017) to 0.114 (±0.037).^19^ However, we caution that Zarzana and co-workers used extinction-only retrievals from CRDS measurements of extinction for mobility-selected particles to constrain their refractive index retrievals for aerosol particles generated from aged BrC samples deriving from the reactions of glyoxal or methylglyoxal with amine precursors. Indeed, a later assessment by the same group was instrumental in arguing for combined measurements of extinction and absorption cross sections for refractive index retrievals and exemplified the large errors in retrieved refractive indices (particularly for *k*) from extinction-only measurements.^33^ The extinction-only retrieval approach is in contrast to our dual-measurement of extinction and absorption cross sections to constrain refractive index retrievals and provides values of up to 0.0016–0.0018. Although the *k* values from our study are not directly comparable to those from Zarzana et al. owing to differences in optical wavelength used and compositions of reaction mixtures,^19^ our UV/vis spectra in Figure 3 demonstrate the *k* values at 532 nm are expected to be negligible (<10^–4^) in comparison to those at 405 nm. Similarly, we may compare our refractive indices with those reported by Zhou et al., who retrieved refractive indices at λ = 532 nm for mobility-selected aerosol particles,^47^ that were generated from aqueous solutions containing 1:1 molar ratios of glyoxal and ammonium sulfate at a pH of ∼ 2.4–3.1, using extinction and scattering cross section measurements. The authors reported unexpectedly large and consistent *k* values of 0.035, at a wavelength for which negligible light absorption is expected for these imine chromophores. Section 1 discusses that these anomalously large values of *k* may be associated with the wavelength and/or truncation correction scheme required for the authors measurements, in addition to the effects of multiple charge artifacts stemming from use of mobility selection.

### 3.4 Effective Densities for Imine BrC Aerosols

We determined the effective densities (ρ_e_) of our generated aerosol particles by measuring their mobility diameters (*d*_m_) after selection on their aerodynamic diameter (*d*_ae_). For spherical and homogeneous particles, the relationship of these two distinct diameter metrics to effective density is given by

$$\rho_{e}=\rho_{0}\frac{d_{\mathrm{ae}}^{2}C_{c}\left(d_{\mathrm{ae}}\right)}{d_{m}^{2}C_{c}\left(d_{m}\right)}$$ 5

in which *C*_c_ is Cunningham slip correction factor (which is a function of diameter) and ρ_0_ is the unit density (1 g cm^–3^). Our determination of particle density using this approach is analogous to that used in our previous work.^39,55^ We control the values of *d*_ae_ via size selection with the AAC to values ranging from 100 to 400 nm in 25 nm intervals. The values of *d*_ae_ that were input to the AAC user interface were corrected by applying a multiplicative calibration coefficient of 0.954 ± 0.011.^39^ The *d*_m_ values were determined from SMPS analysis of the particle mobility diameter distribution, from which the median values of *d*_m_ for the singly charged fraction of the mobility diameter distribution were ascertained by fitting a bimodal log-normal distribution to the measured mobility diameter distribution. Figure S4 shows the regressions of our measurement data, performing a linear regression (forced through the origin) of *d*_ae_^2^ *C*_c_(*d*_ae_) versus *d*_m_^2^ *C*_c_(*d*_m_) with the indicated slopes providing the values of ρ_e_. This regression analysis used the parametrization of the Cunningham slip correction factor reported by Kim and co-workers.^56^

Figure 6 shows the variation in ρ_e_ for our imine BrC aerosol particles with pH_fresh,conc_, in which the error bars represent uncertainties of 5% in the determined ρ_e_; Vokes et al. reported that the retrieved effective densities for nonvolatile aerosol particles from this regression approach were subject to errors of up to ∼ 5%.^55^ The variations in ρ_e_ with pH_fresh,conc_ are reproducible, within their 5% uncertainties, irrespective of whether the particles were generated from freshly prepared solutions or aged solutions that were either in their concentrated or 50-fold diluted forms. Figure 6 also shows ideal mixing predictions of the effective densities for three different assumptions of the particle composition, with all model values calculated using

$$\rho_{e}={\left(\underset{i}{\sum }\frac{w_{i}}{\rho_{i}}\right)}^{-1}$$ 6

in which *w*_i_ and ρ_i_ are the mass fraction and density, respectively, of species *i*, and the summation is over all *i* species comprising the particles. The initial solute concentrations are provided in Table 1. In all calculations, we assumed that the particles were completely dry (after passing through two diffusion driers that reduce the RH of the aerosol-laden sample to <5%) and, in the case of vial K that includes ammonia, that ammonia had evaporated completely from the sampled particles owing to the high vapor pressure of anhydrous ammonia (857 kPa at 293.15 K).^57^ In addition, we assumed that the imine BrC reaction products represented a minor contributor to particle composition and may be ignored. With these assumptions, the mass fractions of species inside the sampled particles were calculated from the concentrations of ammonium sulfate, sulfuric acid, sodium sulfate, and glyoxal in Table 1, and ρ_e_ may then be calculated using eq 6 provided that pure component ρ_i_ values are known. The densities of ammonium sulfate, sulfuric acid, and sodium sulfate are 1.77 g cm^–3^,^58,59^ 1.83 g cm^–3^,^60^ and 2.66 g cm^–3^,^61^ respectively. The density of the glyoxal component is difficult to specify precisely. Glyoxal is unstable in its pure (dry) form and is therefore supplied as a 40% (w/w) solution in which it exists predominantly in its stable dihydrate tetraol form.^14^ On drying, glyoxal oligomerizes and may form oligomeric hydrates even in trace water environments. Therefore, we performed our ideal mixing calculations for three different assumptions of the density associated with the glyoxal component. First (Model 1), we assumed that the glyoxal adopted its monomeric form with a density of 1.14 g cm^–3^.^62^ Alternatively (Model 2), we assumed that the glyoxal component had formed oligomeric hydrates, with the density of glyoxal trimer dihydrate often specified by suppliers as 1.90 g cm^–3^.^63–65^ In addition (Model 3), we treated the density of the glyoxal component as a free parameter that we varied to achieve best agreement with our measured effective densities. From the outset, we acknowledge that the application of the ideal mixing model to these complex organic–inorganic mixtures may not describe mixture densities accurately, particularly for mixtures of organic and inorganic species. For internally mixed particles with known chemical compositions, we reported previously deviations in ideal mixing predictions from measured effective densities for organic–inorganic mixtures of up to 7%.^55^

**Figure 6:**
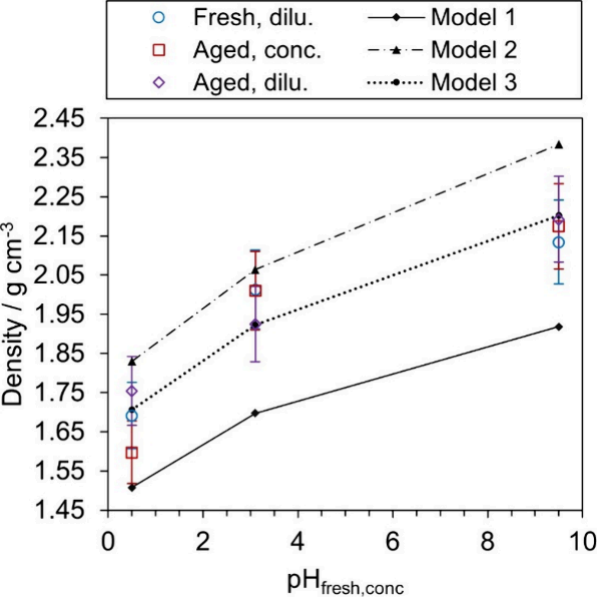
Effective density (ρ_e_) of imine BrC aerosol particles as a function of pH_fresh,conc_. The error bars represent 5% uncertainties in the retrieved effective densities, as described in the main text. Three different ideal mixing model calculations of the material density are also shown, with the main text describing how these calculations were performed. Lines are to guide the eye only.

The Model 1 assumptions lead to significant underpredictions of the densities by ∼ 0.2 g cm^–3^, implying that glyoxal is not present in the dried particles in its monohydrate form. We performed an analogous ideal mixing calculation that additionally assumed the complete evaporation of glyoxal, but these calculations led to drastic overestimates of the effective densities by up to 0.6 g cm^–3^ and are therefore not shown in Figure 6. It is possible for the measured densities to be reconciled by allowing partial evaporation of glyoxal content, although the fraction required for such agreement would vary with pH, requiring 80%, 80%, and 50% of the glyoxal mass to evaporate for vial A, E, and K, respectively. However, such high levels of glyoxal partitioning to the gas phase is unlikely given previous evidence that glyoxal remains predominantly in the condensed phase when aqueous solutions are dried.^66^ Instead, assuming the glyoxal content is retained in oligomeric form with a density identical to that reported for glyoxal trimer dihydrate (1.90 g cm^–3^), the Model 2 ideal mixing model overpredicts the measured densities by up to 0.2 g cm^–3^. This overprediction might arise because the density of the trimer dihydrate reported by suppliers is unreliable or the trimer dihydrate is not formed or forms a variety of oligomeric species. Figure 6 shows that varying the effective density of the glyoxal fraction (Model 3) provided best agreement with the measured densities, with this best-fit density adopting a value of (1.55 ± 0.05) g cm^–3^. This value could be useful for interpreting future studies of dried glyoxal-containing aerosol particles.

## 4 Conclusions

This study provides new high-precision measurements of the complex refractive indices and effective densities of imine brown carbon aerosol particles, that have seen widespread interest, generated from reactions between glyoxal and nitrogen-containing species across a broad pH range. Using an Aerodynamic Aerosol Classifier coupled with cavity ring-down spectroscopy and photoacoustic spectroscopy (both operating at the short visible wavelength of 405 nm), we obtained simultaneous extinction and absorption cross sections that substantially reduce uncertainties in retrieved refractive indices compared to traditional methods that interrogate mobility selected aerosol particles. The AAC-based approach enabled size-resolved optical characterization of low-polydispersity particle populations, improving precision and avoiding multiply charged particle artifacts that undermine mobility-based selection approaches to studying aerosol optical properties.

Our measurements show that detectable visible-light absorption occurs predominantly under basic conditions, corresponding to aerosol particles generated from glyoxal–ammonia mixtures (vial K, pH ≈ 9.5). Under these conditions, the imaginary part of the refractive index, *k* (λ = 405 nm), reached values of 0.0016–0.0018, an order of magnitude lower than prior estimates from extinction-only retrievals yet still sufficient to indicate appreciable light absorption at short visible wavelengths. Aerosols generated under acidic and near-neutral conditions (vials A and E) exhibited negligible absorption at 405 nm, consistent with the absence of visible-light-absorbing chromophores in corresponding bulk UV/vis absorption spectra. Comparison of aerosol and bulk-phase measurements suggests that aerosolization and subsequent drying enhance the formation of light-absorbing species, likely through increased solute concentrations during water evaporation that facilitate imine polymerization in metastable, supersaturated states. These findings reinforce the importance of unique aerosol processing in determining the optical evolution of BrC in the atmosphere. Comparison with previous laboratory studies^19,47^ highlights that overestimation of *k* values in earlier work likely arose from methodological limitations such as multiple-charge artifacts and inaccurate scattering truncation and wavelength-dependent corrections. Effective particle densities determined from combined aerodynamic and mobility sizing were in the range (1.60 – 2.19) g cm^–3^, consistent with partially oligomerized glyoxal-containing mixtures and supporting the conclusions of previous work on the predominance of oligomeric hydrate species within dried glyoxal particles. Ideal mixing calculations suggest that the measured densities are reproduced best when the glyoxal fraction is assigned an effective density near 1.55 g cm^–3^.

Chromophore formation under basic conditions contributes modest absorption at visible wavelengths, implying a limited contribution of imine BrC to atmospheric aerosol direct radiative effects compared to more strongly absorbing BrC classes (e.g., nitroaromatics),^67^ particularly given that the pH of typical ambient aerosol and cloud droplets correspond to acidic and near-neutral conditions. Future studies should extend measurements across broader spectral ranges and relative-humidity conditions to quantify wavelength-dependent absorption and assess aging effects.

## Supplementary Material



## Notes

UV/vis spectra for imine BrC bulk solutions. Measured versus modeled optical cross sections. Aerodynamic versus mobility particle diameter analysis for density determination. Table of retrieved values for aerosol particle refractive indices (PDF)
