# Supplementary material for: Spectroscopic Analysis of the Complex Refractive Indices for Imine Brown Carbon Aerosol Particles
Source: ACS Earth Space Chem. 2026 Feb 12;10(3):897–907. doi: 10.1021/acsearthspacechem.5c00392 (PMC13007030; doi:10.1021/acsearthspacechem.5c00392)
Supplement: Supplementary file 1 [file sp5c00392_si_001.pdf]

Supporting Information for "Spectroscopic Analysis of the Complex Refractive Indices for Imine Brown Carbon Aerosol Particles"

Simon Xi Chen,[1] Gwen R. Lawson,[1] James D. Allan,[2,3] Justin M. Langridge,[4] and Michael I. Cotterell[5]*

[1]School of Chemistry, University of Bristol, Bristol, UK, BS8 1TS

[2]Department of Earth and Environmental Sciences, University of Manchester, Manchester, UK, M13 9PL

[3]National Centre for Atmospheric Science, The University of Manchester, Manchester, UK, M13 9PL

[4]Met Office, Exeter, UK, EX1 3PB

[5]Department of Chemistry, University of Oxford, Oxford, UK, OX1 3QZ

* To whom correspondence should be addressed: michael.cotterell@chem.ox.ac.uk

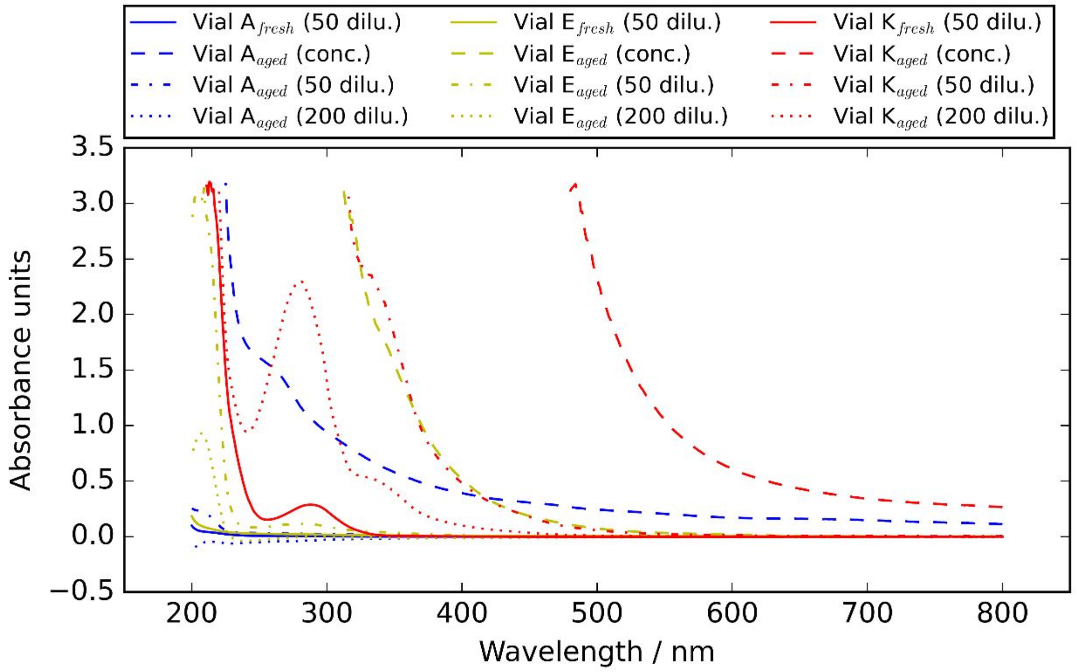

Figure S1. UV/Vis spectra for the 50-fold diluted fresh solutions, and corresponding spectra for the aged solutions under concentrated, 50-, and 200-fold dilution conditions solutions. Spectra are shown for vials A, E, and K.

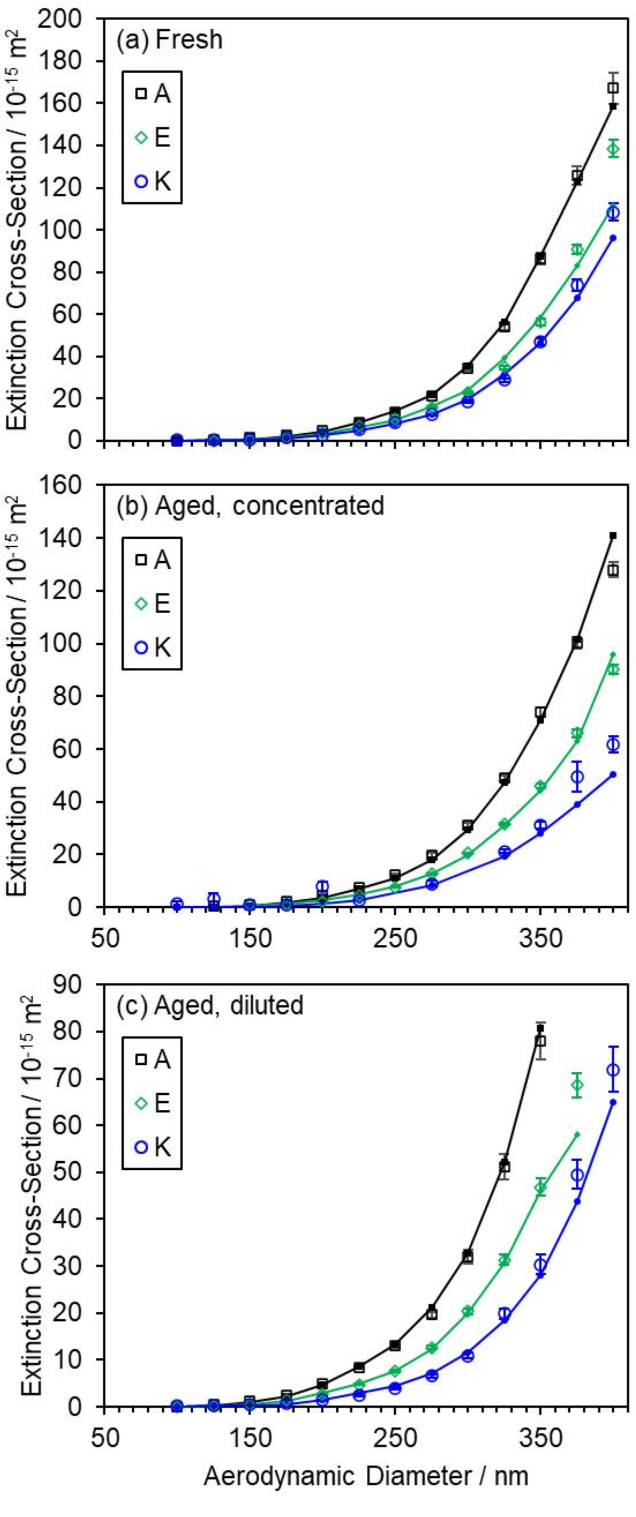

Figure S2. The measured and modelled extinction cross-sections with variation in the selected aerodynamic diameter for aerosol particles generated from (a) fresh solutions, (b) aged and concentrated solutions, and (c) aged and diluted solutions. Open symbols denote measured values, and filled symbols denote the best-fit cross-sections from Lorenz-Mie theory. Error bars represent one standard deviation in the measured cross-sections. Lines are to guide the eye only for the best-fit modelled cross-sections. Black, green, and blue data points correspond to Vial A, E, and K, respectively, as indicated in the panel legends.

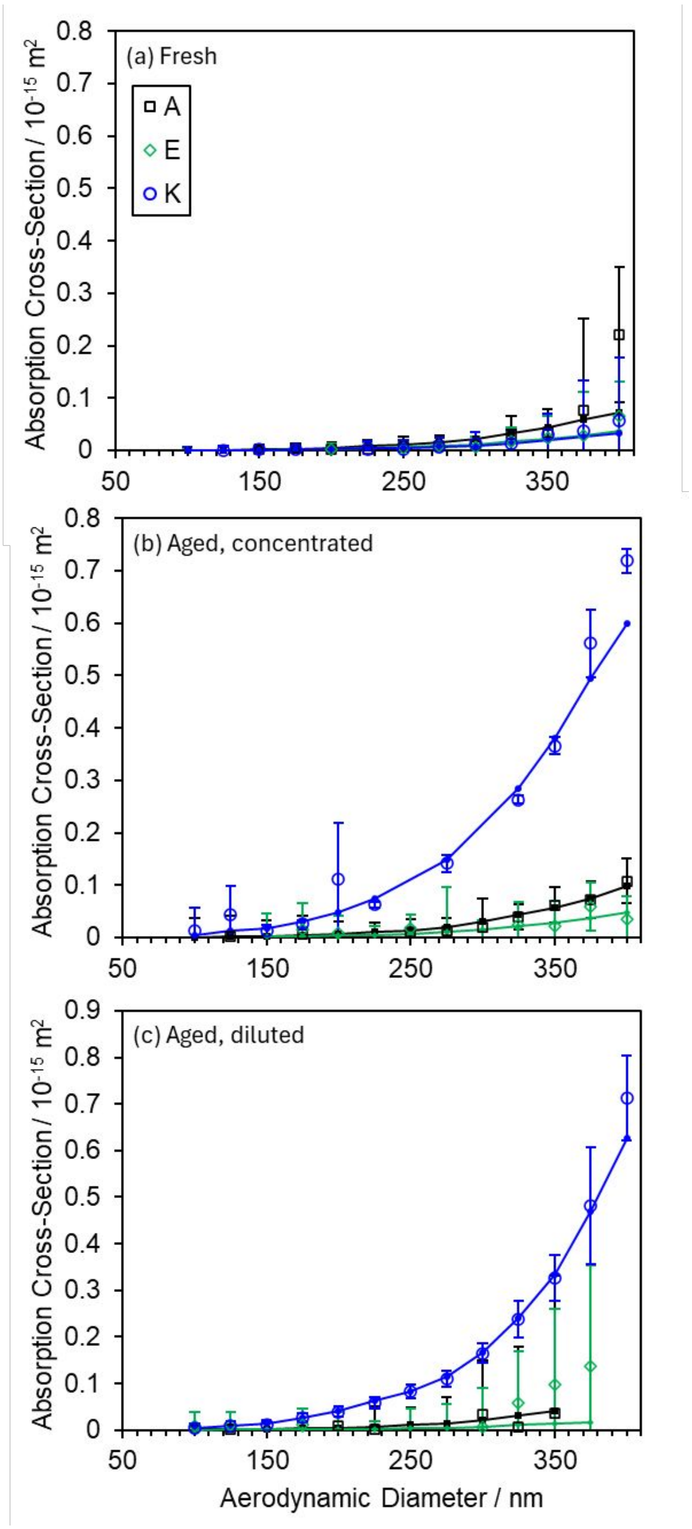

Figure S3. The measured and modelled absorption cross-sections with variation in the selected aerodynamic diameter for aerosol particles generated from (a) fresh solutions, (b) aged and concentrated solutions, and (c) aged and diluted solutions. Open symbols denote measured values, and filled symbols denote the best-fit cross-sections from Lorenz-Mie theory. Error bars represent one standard deviation in the measured cross-sections. Lines are to guide the eye only for the best-fit modelled cross-sections. Black, green, and blue data points correspond to Vial A, E, and K, respectively, as indicated in the panel legend.

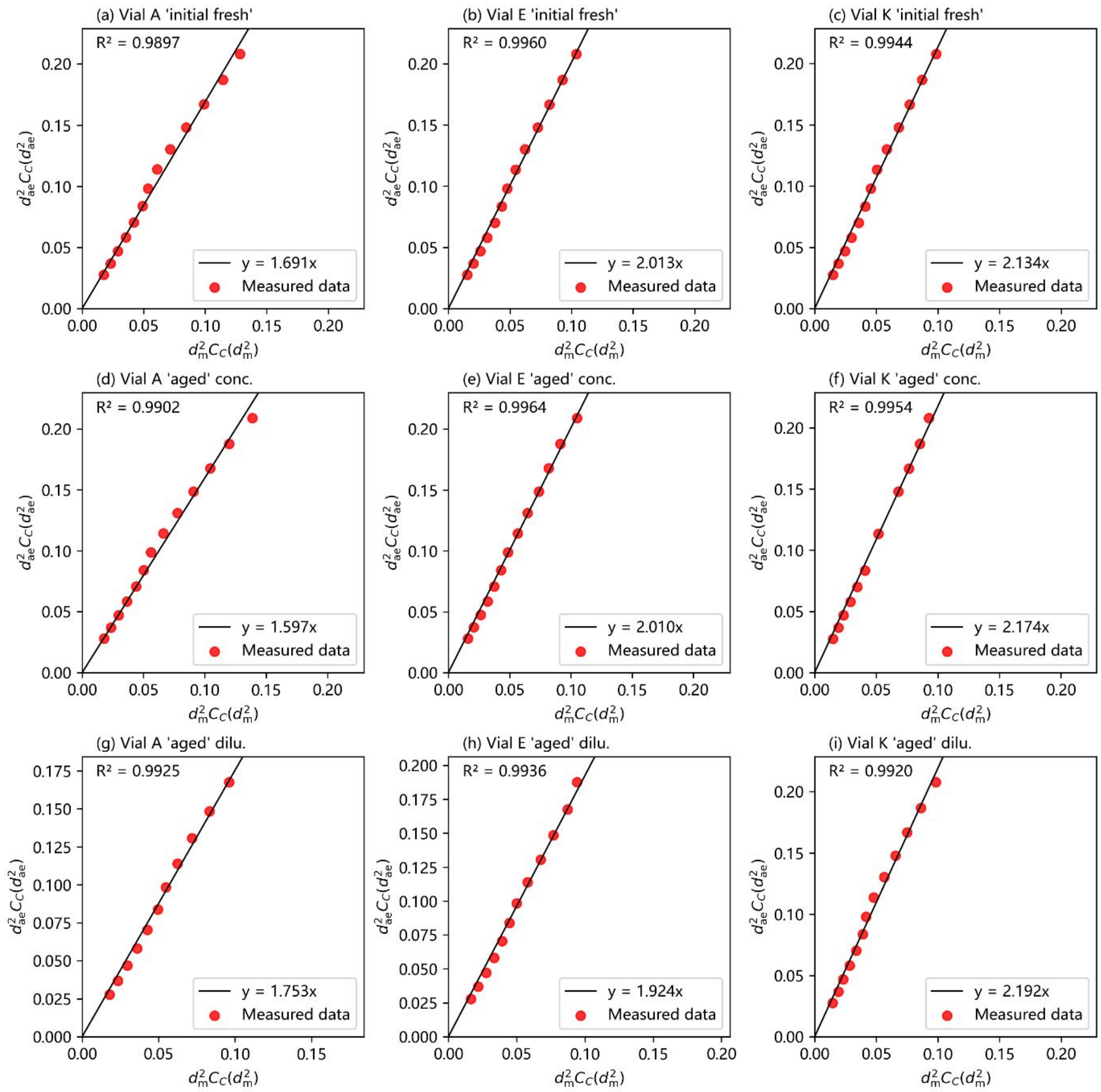

Figure S4. Plots of $d_{\mathrm{ae}}^2 C_{\mathrm{c}}(d_{\mathrm{ae}})$ vs $d_{\mathrm{m}}^2 C_{\mathrm{c}}(d_{\mathrm{m}})$, with the best-fit of a straight line through the measured distributions shown. The coefficient of determination for these linear fits is indicated.

Vial	n	k
$\mathbf{A}_{\mathbf{fresh,dilu}}$	1.5368	0.0001
$\mathbf{E}_{\mathbf{fresh,dilu}}$	1.5788	0.0001
$\mathbf{K}_{\mathbf{fresh,dilu}}$	1.5718	0.0001
$\mathbf{A}_{\mathbf{aged,conc}}$	1.4911	0.0000
$\mathbf{E}_{\mathbf{aged,conc}}$	1.5315	0.0001
$\mathbf{K}_{\mathbf{aged,conc}}$	1.4751	0.0018
$\mathbf{A}_{\mathbf{aged,dilu}}$	1.5456	0.0001
$\mathbf{E}_{\mathbf{aged,dilu}}$	1.4818	0.0000
$\mathbf{K}_{\mathbf{aged,dilu}}$	1.4719	0.0016

Table S1. The refractive indices (RI, $m = n + ik$) of imine bulk solutions.
